# The roles of movement and coat proteins in the transport of tobamoviruses between plant cells

**DOI:** 10.3389/fpls.2025.1580554

**Published:** 2025-04-17

**Authors:** Yumin Kan, Vitaly Citovsky

**Affiliations:** Department of Biochemistry and Cell Biology, State University of New York, Stony Brook, NY, United States

**Keywords:** tobamovirus, phylogenic analysis, cell-to-cell movement, plasmodesmata, systemic spread

## Abstract

*Tobamovirus* is a large group of positive-sense, single-stranded RNA viruses that cause diseases in a broad range of plant species, including many agronomically important crops. The number of known *Tobamovirus* species has been on the rise in recent years, and currently, this genus includes 47 viruses. Tobamoviruses are transmitted mainly by mechanical contact, such as physical touching by hands or agricultural tools; and some are also transmitted on seeds, or through pollinator insects. The tobamoviral genome encodes proteins that have evolved to fulfill the main conceptual task of the viral infection cycle - the spread of the invading virus throughout the host plant cells, tissues, and organs. Here, we discuss this aspect of the infection cycle of tobamoviruses, focusing on the advances in our understanding of the local, i.e., cell-to-cell, and systemic, i.e., organ-to-organ, virus movement, and the viral and host plant determinants of these processes. Specifically, we spotlight two viral proteins—the movement protein (MP) and the coat protein (CP), which are directly involved in the local and systemic spread of tobamoviruses—with respect to their phylogeny, activities during viral movement, and interactions with the host determinants of the movement process.

## Introduction

With climate change and a rapidly growing human population, the demand for global food production will become a main challenge in the near future (Jones and Naidu, 2019; Cassman and Grassini, 2020). Plant viruses comprise about 50% of pathogens that infect plants worldwide, causing economic losses of more than US$30 billion annually (Jones and Naidu, 2019). The number of known species of *Tobamovirus*, which belongs to the Virgaviridae family of positive-strand RNA viruses, has been increasing, and currently, this genus contains 47 species (10 of which are not yet recognized by the International Committee on Taxonomy of Viruses, ICTV, as species; **Table 1**) (ICTV, 2024). Tobamoviruses comprise several subgroups according to their host plants, including Solanaceae, Cucurbitaceae, and Brassicaceae, which are represented by tobacco mosaic virus (TMV), cucumber green mottle mosaic virus (CGMMV), and turnip vein clearing virus (TVCV), respectively (Lartey et al., 1997; Dombrovsky et al., 2017; Li et al., 2017). Tobamoviruses are also known for their pathogenic effects on crops; for example, tomato brown rugose fruit virus (ToBRFV) and CGMMV cause diseases of agronomically important crops with global and local significance (Tatineni and Hein, 2023). It is important to note that the host range, although serving as a convenient criterion for grouping tobamoviruses, depends on many factors, from the genetic determinants of the virus-host combinations to the environmental factors, and, therefore, is usually relatively wide (Zamfir et al., 2023).

**Table 1:** *Tobamovirus* species and the disease symptoms they cause on plants.

No.	Host species	Sub No.	Species	Host	Disease symptom	Ref.
1	Solanaceae	17-1	Bell pepper mottle virus (BPeMV)	Pepper crops	Necrotic local lesions; systemic necrotic infection; mosaic or mottle	(Wetter et al., 1987)
2		17-2	Brugmansia latent virus (BrLV)	Brugmanisa plant	No obvious viral symptoms	(Ilyas et al., 2022)
3		17-3	Brugmansia mild mottle virus (BMMV)	*Brugmansia* sp.	Systemically; mild mottling; vein necrosis	(Ilmberger et al., 2007)
4		17-4	Chili pepper mild mottle virus (CPMMV)	Pepper	——	(Ontañón et al., 2024)
5		17-5	Obuda pepper virus (ObPV)	Pepper	Local necrotic lesions	(Kalapos et al., 2021)
6		17-6	Paprika mild mottle virus (PaMMV)	Pepper	Mosaic on leaves; mottle/circular pitting on fruits	(Hamada et al., 2003)
7		17-7	Pepper mild mottle virus (PMMoV)	*Capsicum* spp.	Leaves: deformed, mild chlorotic or no foliar symptoms; mottled leaves; vein thickening; Fruits: mosaic; deformations; Stunting	(Kalapos et al., 2021; Kumari et al., 2023)
8		17-8	Scopolia mild mottle virus (SMMoV)	*Scopolia japonica*	Mild mottle on upper leaves	(Uehara-Ichiki et al., 2022)
9		17-9	Tobacco latent virus (TLV)	Tobacco	No obvious symptoms	(Ilyas et al., 2022)
10		17-10	Tobacco mild green mosaic virus (TMGMV)	Plants in Solanaceous, Apocynaceae, Chenopodiaceae, Balsaminaceae, Commelinaceae, Gesneriaceae, Compositae and Umbelliferae family	Chlorotic, mosaic or mottling, distortion and surface reduction of leaves; irregular shapes and colour of fruits; Necroses on leaves and fruits	(Zhao et al., 2021)
11		17-11	Tobacco mosaic virus (TMV)	Solanaceae and many species outside of the Solanaceae	Mosaic on leaves	(Scholthof, 2004, 2023)
12		17-12	Tomato brown rugose fruit virus (ToBRFV)	Tomato and pepper	Mosaic, deformation, and necrosis on tomato leaves; puckering, yellow mottling on pepper leaves, yellow to brown rugose dots and necrosis on fruits, stunting on pepper seedlings	(Zhang et al., 2022)
13		17-13	Tomato mosaic virus (ToMV)	Tomato	Mosaic, curling, and distortion on leaves; internal browning and uneven ripening on fruits	(Ullah et al., 2017)
14		17-14	Tomato mottle mosaic virus (ToMMV)	Tomato and pepper	Necrosis and chlorosis and deformation on leaves; necrosis on fruit	(Li et al., 2017; Sui et al., 2017)
15		17-15	Tropical soda apple mosaic virus (TSAMV)	Solanaceae and Chenopodiaceae plants	Foliar mosaic	(Adkins et al., 2007)
16		17-16	Yellow pepper mild mottle virus	——	——	——
17		17-17	Yellow tailflower mild mottle virus (YTMMV)	Plants in solanaceous species	Inter-veinal clearing, leaf and flower deformation, with dark-green islands on leaves	(Wylie et al., 2014)
18	Cucurbitaceae	7-1	Cucumber fruit mottle mosaic virus (CFMMV)	Melon, cucumber, pumpkin	Severe mottling or mosaic on cucumber fruits; fruit distortion; leaf mosaic and necrotic lesions on fruit	(Antignus et al., 2001)
19		7-2	Cucumber green mottle mosaic virus (CGMMV)	Cucurbit crops	Systemic green mottle mosaic on foliage	(Mandal et al., 2008; Dombrovsky et al., 2017)
20		7-3	Cucumber mottle virus (CuMoV)	Several species of cucurbits	Mild mottling on leaves; mosaic symptoms on fruit and leaves	(Orita et al., 2007)
21		7-4	Kyuri green mottle mosaic virus (KGMMV)	Cucurbitaceous vegetable crops	Fruit distortion; mottle mosaic	(Yoon et al., 2001)
22		7-5	Trichosanthes mottle mosaic virus (TrMMV)	*Trichosanthes kirilowii*	Mottling and mosaic symptoms	(Chen et al., 2022)
23		7-6	Watermelon green mottle mosaic virus (WGMMV)	Cucurbit crops	CGMMV-like symptoms; mottle, mosaic and leaf crinkling	(Cheng et al., 2019; Pitman et al., 2019)
24		7-7	Zucchini green mottle mosaic virus (ZGMMV)	Zucchini squash plants	Foliar mottle and severe mosaic; abnormal fruits	(Ryu et al., 2000)
25	Brassicaceae	3-1	Turnip vein-clearing virus (TVCV)	Crucifer family plants	Vein-clearing on turnips	(Melcher, 2003)
26		3-2	Wasabi mottle virus (WMoV)	Wasabi plants	Ringspots, vein-clearing	(Macdonald et al., 2021)
27		3-3	Youcai mosaic virus (YoMV), also known as TMV-Cg, Chinese rape mosaic virus (CRMV), or Oilseed rape mosaic virus (ORMV)	Alstroemeriaceae, Asteraceae, Balsaminaceae, Brassicaceae, Gentianaceae, Liliaceae, Orobanchaceae, and Solanaceae	Mosaic symptoms	(Fuji et al., 2007; Ju et al., 2019)
28	Apocynaceae	4-1	Frangipani mosaic virus (FrMV)	Frangipani plants	Mosaic symptoms on leaves	(Choliq et al., 2017; Kumar et al., 2022)
29		4-2	Hoya chlorotic spot virus (HoCSV)	Hoya plants	Chlorotic spots, ringspots, and ring patterns	(Adkins et al., 2018)
30		4-3	Hoya necrotic spot virus	Hoya plants	Necrotic foliar lesions	(Ilyas et al., 2021)
31		4-4	Plumeria mosaic virus (PluMV)	Frangipani plants	Mosaic symptoms on leaves	(Kumar et al., 2022)
32	Cactaceae	5-1	Cactus mild mottle virus (CMMoV)	Cactus plants	Mild mottle on stem	(Min et al., 2006)
33		5-2	Cactus tobamovirus 1	——	——	—
34		5-3	Cactus tobamovirus 2	——	——	—
35		5-4	Opuntia virus 2 (OV2)	Prickly pear plant	——	(Salgado-Ortíz et al., 2020)
36		5-5	Rattail cactus necrosis-associated virus (RCNaV)	Cactus plants	Necrosis symptoms	(Kim et al., 2012)
37	Leguminosae	2-1	Clitoria yellow mottle virus (CYMV)	*Clitoria ternatea*	Local chlorotic lesions, systemic vein clearing, mottling and mosaic of the tip leaves	(Wei et al., 2012)
38		2-2	Sunn-hemp mosaic virus (SHMV)	Leguminous plants	Mosaic, puckering, blistering, malformation on leaves; plant stunting	(Varma, 1986)
39	Malvaceae	2-1	Hibiscus latent Fort Pierce virus (HLFPV)	Hibiscus and related species	Chlorotic spots and chlorotic mottling	(Allen et al., 2005)
40		2-2	Hibiscus latent Singapore virus (HLSV)	Hbiscus and related species	Chlorotic spots and chlorotic mottling	(Allen et al., 2005)
41	Passifloraceae	2-1	Maracuja mosaic virus (MarMV)	Passion fruit	Systemic mosaic on *N. benthamiana*, and local lesions on *N. tabacum*, *N. glutinosa* and *Gomphrena globosa*	(Song and Ryu, 2011)
42		2-2	Passion fruit mosaic virus (PafMV)	Passion fruit	——	(Song and Ryu, 2011)
43	Basellaceae	1-1	Ullucus mild mottle virus (UMMV)	*Ullucus tuberosus*	Symptomless or inconspicuous symptom	(Brunt et al., 1982)
44	Gesneriaceae	1-1	Streptocarpus flower break virus (SFBV)	*Streptocarpus* plants	Necrotic rings	(Tang et al., 2024)
45	Orchidaceae	1-1	Odontoglossum ringspot virus (ORSV)	Orchids; also infect Solanaceae and Chenopodiaceae	Cymbidium diamond mottle, infectious blossom Necrosis, Cattleya mild flower break, and Cattleya color break	(Edwardson and Zettler, 1986; Rabindran et al., 2005)
46	Orobanchaceae	1-1	Rehmannia mosaic virus (ReMV)	Rehmannia, Solanaceae	Necrotic mosaic	(Kubota et al., 2012)
47	Plantaginaceae	1-1	Ribgrass mosaic virus (RMV)	Plantain, Brassicaceae	Interveinal mottling	(Kim et al., 2010; Chavan and Pearson, 2016)

1, the species with underlined names are not recognized by the ICTV as species; 2, Opuntia chlorotic ringspot virus: a synonym for the Sammons opuntia virus, for which two sequences were obtained, designated cactus tobamovirus 1 and cactus tobamovirus 2.

Tobamoviruses are mainly transmitted by mechanical contact, and recent studies suggest that at least some tobamoviruses, such as TMV, ToBRFV, and CGMMV, can also be transferred by insect vectors (Okada et al., 2000; Darzi et al., 2018; Levitzky et al., 2019) as well as through seeds (Dombrovsky and Smith, 2017). Disease symptoms of the tobamoviral infection include leaf deformation or mosaic, mottled fruit, systemic necrosis, or defoliation (Ilyas et al., 2022; Spiegelman and Dinesh-Kumar, 2023), although infection of some plant species may also cause no obvious symptoms (Ilyas et al., 2022). Among all plant viruses, TMV was the first virus identified and chemically purified more than one century ago (Creager et al., 1999; Spiegelman and Dinesh-Kumar, 2023), laying the foundation for the science of virology.

The *Tobamovirus* genus comprises a group of viruses with positive-sense, single-stranded RNA (+ssRNA) genomes of approximately 6.4 kb in length. The tobamoviral genome usually contains four open reading frames (ORFs) that produce four proteins: two replicase-associated proteins, 126-kDa and 183-kDa, which share the same start codon and can produce the 183-kDa protein when translated by the read-through of the amber codon of the 126-kDa-encoding ORF; a cell-to-cell movement protein (MP); and a coat (or capsid) protein (CP) (Spiegelman and Dinesh-Kumar, 2023). Both MP and CP genes are transcribed from their own subgenomic promoters (Grdzelishvili et al., 2000). During infection, the four tobamoviral proteins are expressed differentially. Specifically, among the viral replication complex (VRC) proteins, MP is expressed and accumulates in VRCs mainly at the early infection stage, whereas CP is expressed and becomes associated with VRCs at the late infection stages (Asurmendi et al., 2004). These observations are consistent with the previous studies that ca. 12 hours after infection, the VRCs, containing the replicase, viral RNA, and MP, were assembled at the cortical endoplasmic reticulum. After 12-16 hours, these VRCs move intracellularly until they reach PD, followed by translocation through PD to the adjacent cells after 18-20 hours. In the newly infected cells, this process is repeated about every 24 hours (Kawakami et al., 2004). Interestingly, MP expression and VRC formation are positively regulated by CP later in the infection, whereas premature expression of CP decreases cell-to-cell movement and viral infection (Kawakami et al., 2004).

Some *Tobamovirus* species also contain an additional small ORF, designated ORF6 and coding for a 4.8-kDa protein, that overlaps the MP ORF and CP ORF (Morozov et al., 1993; Ishibashi and Ishikawa, 2016). There is also a 54-kDa protein, detected only using *in vitro* translation, which contains the RNA-dependent RNA polymerase domain (Palukaitis et al., 2024). However, the functions of the 4.8-kDa or 54-kDa proteins remain unclear.

The *Tobamovirus* genome organization and the activities of its encoded proteins have evolved to ensure the optimal spread of the invading virions throughout the host plant cells, tissues, and organs. This aspect of the infection cycle of tobamoviruses represents the focus of our review which discusses the fundamentals and recent advances in our understanding of the local, i.e., cell-to-cell, and systemic, i.e., organ-to-organ, virus movement and the viral and host plant determinants of these processes.

## Viral determinants of local and systemic movement

The movement of plant viruses within their host plants comprises the local, i.e., cell-to-cell, transport and the long-distance, i.e., systemic, transport. The local movement of the virus begins following the initial infection, translation, and replication. This cell-to-cell spread from the initially infected cells occurs through the intercellular connections, the plasmodesmata (PD), presumably in the form of subviral particles. When the moving virus encounters the host vascular system, it enters the vasculature and commences the systemic spread that delivers the virus to the uninfected plant organs, in which it exits the vascular tissues and spreads locally. That CP is needed for viral entry into the vasculature (Solovyev and Savenkov, 2014) suggests that fully or partially encapsidated virus particles are involved in this transport process.

### Cell-to-cell movement and movement protein

Unlike some plant viruses that may not rely on MP for their intercellular movement (Ying et al., 2024), tobamoviruses require the participation of MP, the function of which was identified almost four decades ago, for the cell-to-cell spread (Deom et al., 1987), but the mechanism by which MP performs this function remains largely unresolved to this day. Tobamoviral MP can bind single-strand nucleic acids cooperatively and sequence-nonspecifically, presumably facilitating the transport of the viral genomes through PD (Citovsky et al., 1990). Virions of tobamoviruses, such as TMV, are rigid 18×300 nm rod-shaped particles that normally do not cross the PD channels, i.e., cytoplasmic sleeves through which most of the soluble molecule movement occurs, that are ca. 10-nm wide (Oparka et al., 1999; Tee and Faulkner, 2024). This process is thought to be mediated by the viral MP, which associates with and protects the genome cargo (Citovsky et al., 1992) and sorts to PD, increasing their permeability (Wolf et al., 1989; Citovsky et al., 1992; Waigmann et al., 1994) to allow the transport of the viral genomes and their cognate proteins. MP increases the PD molecular size exclusion limit (SEL) by targeting the host callose homeostasis at PD to reduce callose deposits (Culver and Padmanabhan, 2007; De Storme and Geelen, 2014), including those induced by dsRNA signaling, an important defense response against virus infection (Huang et al., 2023).

Within the tobamoviral MP molecule, several domains and specific amino acid residues are important for the subcellular localization and cell-to-cell movement function. For example, in TMV MP, the N-terminal 50 amino acids, especially the valine at the fourth position and the phenylalanine at position 14, are critical for the plasmodesmal localization, and their deletion or substitution, respectively, results in a loss of PD localization and accumulation of the mutant MP in the cell cytoplasm and nucleus (Yuan et al., 2016). Also, the C-terminal amino acid residues between positions 126 and 224 are involved in the regulation of the PD SEL (Waigmann et al., 1994), the amino acid residues between positions 130 and 185 are involved in the TMV MP interaction with the host pectin methylesterases and are required to increase the PD SEL and sustain the cell-to-cell movement (Waigmann et al., 1994; Chen et al., 2000), and the C-terminal amino acid residues between positions 112-185, and between 185-268 are required for binding of single-strand nucleic acids and cell-to-cell movement (Waigmann et al., 1994). In addition, the serine residue at position 37 of ToMV MP is essential for protein stability and localization (Kawakami et al., 1999).

**Figure 1:**
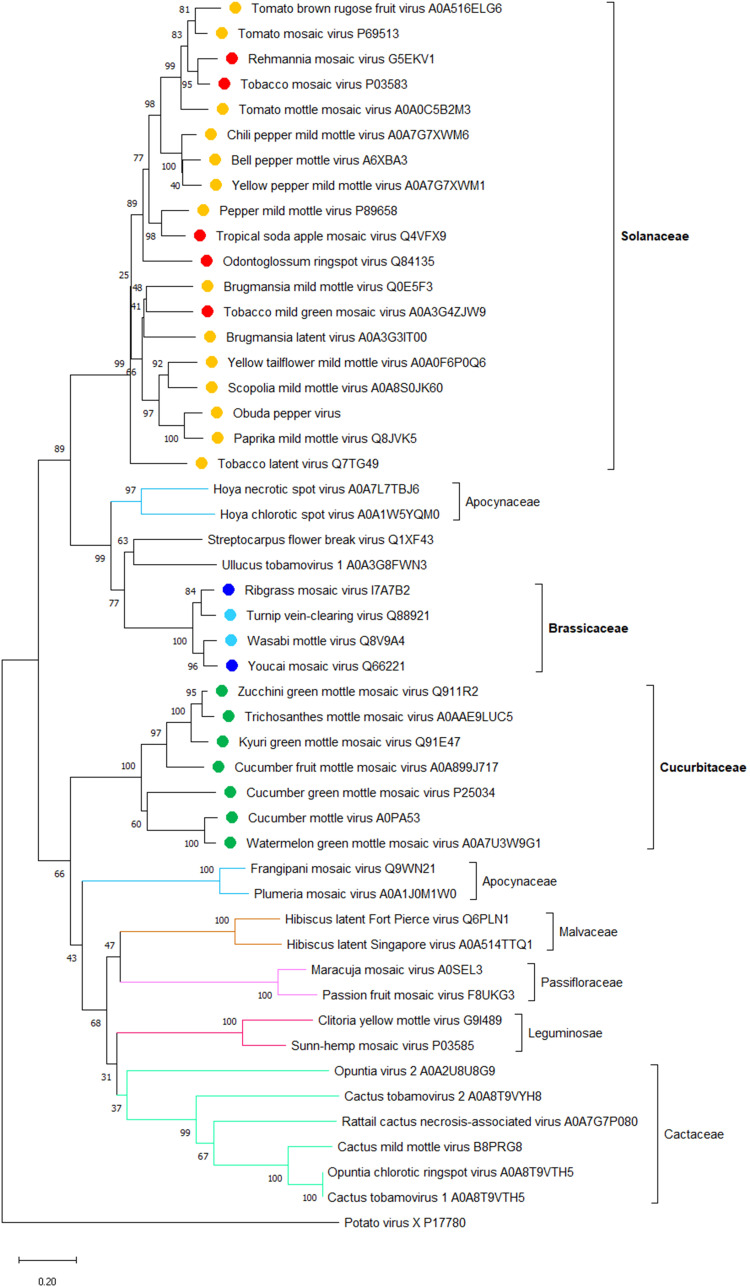
**A** phylogenetic analysis of amino acid sequences of movement proteins (MP) from 47 species of the *Tobamovirus* genus. Potato virus X MP was used as the out-group sequence. MPs are shown relative to the families of the host plants. MPs marked with orange, light blue, and dark green are encoded by the viruses that primarily infect host plants of Solanaceae, Brassicaceae, and Cucurbitaceae, respectively. MPs marked with red and dark blue are encoded by the viruses that can also infect plants in additional families besides Solanaceae and Brassicaceae, respectively. The neighbor-joining (NJ) method was adopted using MEGA software (version 10.1.8) with 1,000 bootstrap replicates. The trees are drawn to scale, with branch lengths measured in the number of substitutions per site. Scale bars, 0.20 amino acid substitutions per site. The individual MP sequences are identified by the name of the virus and the corresponding Protein ID.

The amino acid sequence of MP varies among taxonomic groups of plant viruses, and different MPs often exhibit different types of cell-to-cell transport (Taliansky et al., 2008), e.g., TMV MP likely transports the viral RNA through PD as a ribonucleoprotein complex (Citovsky et al., 1990), whereas MP of the cauliflower mosaic virus (CaMV) forms trans-PD tubules through which the virions are translocated (Kasteel et al., 1996). In the CLANS analysis of amino acid sequences of 389 MPs from different virus families, 16 clusters were detected, and the tobamoviral MPs fell into the same cluster (Butkovic et al., 2023). The genomes of all known species of tobamoviruses encode a single MP belonging to the 30K protein superfamily and ranging in size from 27.9 kDa (opuntia virus 2) to 38.9 kDa (cactus tobamovirus 2). Here, we examined a possible phylogenetic relationship between tobamoviruses’ relatively diverse MP sequences and their natural host plant preferences. We randomly selected one strain from each of all tobamoviral species with available genomic information and analyzed their phylogeny using MP of an unrelated potato virus X as the out-group sequence. **Figure 1** shows that the MP amino acid sequences formed clades according to their host plants. For example, 17 viruses that infect Solanaceae, seven viruses that infect Cucurbitaceae, and three viruses that infect Brassicaceae fall into three distinct groups, according to the main host plant family (**Figure 1**, **Table 1**), suggesting a role for tobamoviral MP sequences in the determination of the viral host range. Potentially, the subcellular localization of MPs may differ between tobamoviruses belonging to different clades. For example, MP of TMV localizes almost exclusively to PD in the host cell, whereas MP of TVCV sorts both to PD and the cell nucleus in *Nicotiana benthamiana* and *Arabidopsis thaliana* (Levy et al., 2013). In addition, tobamoviral MPs may determine interactions with different host genotypes, such as the case where MP of ToBRFV, but not of TMV or ToMV, can break the durable resistance of the tomato *Tm-2^2^* genotype (Hak and Spiegelman, 2021; Yan et al., 2021).

Although MP is the main effector of cell-to-cell transport, the 126-kDa replicase-associated protein also plays a role in this process. For example, TMV mutants in the helicase-like domain of TMV replicase retain the replication ability yet no longer move between cells (Hirashima and Watanabe, 2001); the role of the 126-kDa replicase-associated protein in the local movement was suggested to involve the non-conserved region of the protein (Hirashima and Watanabe, 2003). Similarly, the 122-kDa replicase of a crucifer-infecting TMV strain was shown to have a silencing suppressor activity (Csorba et al., 2007). The role of viral replicase in intercellular transport is lent additional support by the observations that this movement involves the replication complexes of the virus (Kawakami et al., 2004; Wu and Cheng, 2020); the precise contribution of this co-replication to the overall viral cell-to-cell transport remains to be elucidated. In addition, the methyltransferase and helicase domains, as well as the non-conserved region II (NONII), of the 126-kDa replicase-associated protein, each functions as a suppressor of the RNA silencing (Wang et al., 2012).

### Systemic movement and coat protein

Tobamoviruses traffic long-distance through the host phloem in the source-to-sink direction together with the photoassimilates (Hipper et al., 2013). This systemic mode of viral movement begins with the invasion of the minor veins (Ding et al., 1998), which occurs once the virus has spread cell-to-cell from the inoculated epidermis to the mesophyll, phloem parenchyma, and companion cells and entered the phloem (Carrington et al., 1996). Importantly, whereas the cell-to-cell movement of tobamoviruses absolutely depends on MP, their systemic movement requires the viral CP (Solovyev and Savenkov, 2014). That may be due to the critical role of CP in the viral assembly (Saito et al., 1990), although MP also appears to participate in the long-distance movement by as yet unknown mechanism, which is different from the MP activity during the cell-to-cell movement (Fenczik et al., 1995). Because the cell-to-cell movement activity of MP is a prerequisite for systemic transport, the precise role of MP in the latter process is difficult to elucidate. This role, however, is consistent with the recent isolation of a spontaneous MP mutant that lacks the 16 C-terminal amino acids and allows long-distance movement of a CP-deficient TMV strain (Tran et al., 2022). Thus, tobamoviral MPs likely possess an evolutionary potential to gain a new protein function that facilitates long-distance movement of viral genomes in the absence of the natural viral mediator, i.e., CP, of this movement. This is in agreement with the hypothesis—based on the sequence homology between the core structural domain of MPs and the jelly-roll domain of CPs of small viruses with icosahedral capsids—that tobamoviral MPs have evolved from duplication of the single jelly-roll CP gene (Butkovic et al., 2023).

Based on the requirement of CP and MP for the local and systemic movement, plant viruses can be classified into three types: in type I, CP is not required for cell-to-cell movement; in type II, CP plays a similar role to MPs; and the type III viruses move as viral particles, requiring CP for movement (Scholthof, 2005). Tobamoviruses utilize the type I movement mechanism (Scholthof, 2005). Indeed, CP deletion mutants of TMV lost the systemic transport ability, although the virus could reach the bundle sheath and parenchyma cells by cell-to-cell movement (Solovyev and Savenkov, 2014). Interestingly, the ability of CP to promote systemic movement may be species-specific among different tobamoviruses. For example, CP of TMV cannot be substituted by CP from another tobamovirus, odontoglossum ringspot virus (ORSV), whereas the cell-to-cell movement of such chimeric viruses was not affected (Hilf and Dawson, 1993). The molecular mechanism by which CP facilitates viral movement is still obscure, but recent evidence suggests that it extends beyond mere viral assembly and may involve interactions with the host plant’s innate immunity. Specifically, the role of CP in the systemic movement of TMV was shown to involve the downregulation of the salicylic acid (SA) pathway, and this regulation was proposed to occur via the stabilization of the DELLA proteins by an as yet unknown process (Venturuzzi et al., 2021).

**Figure 2:**
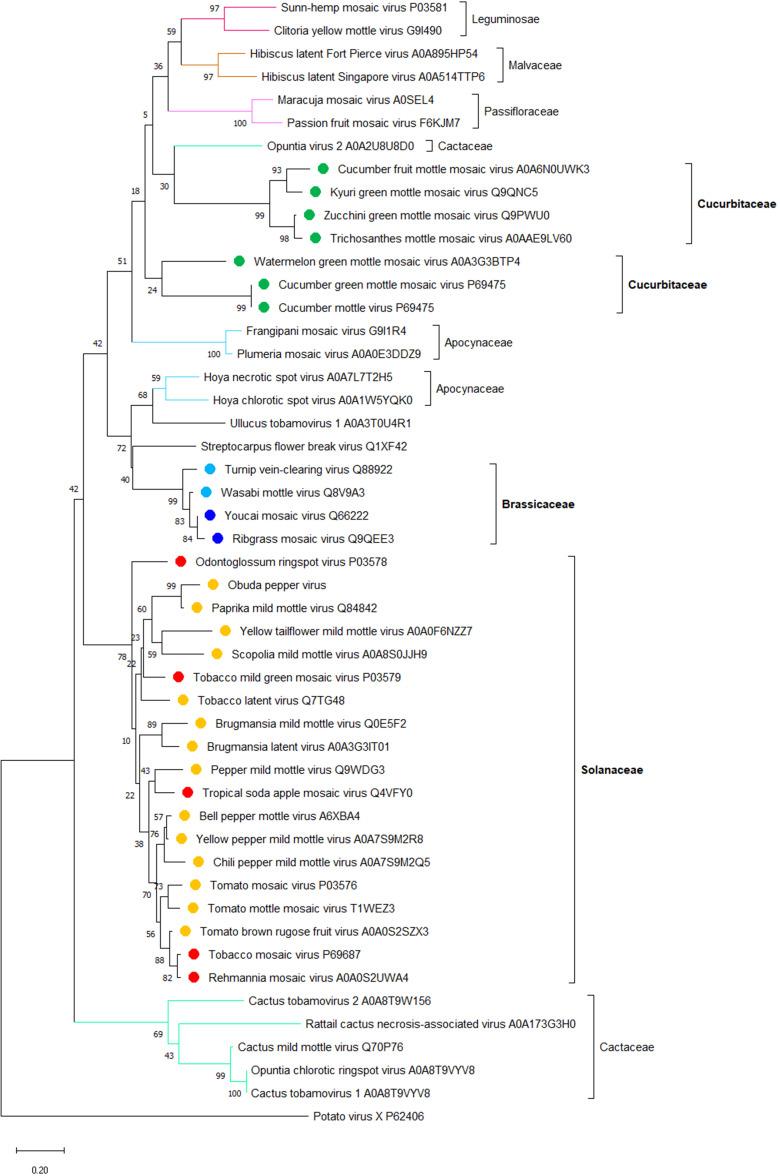
**A** phylogenetic analysis of amino acid sequences of coat proteins (CP) from 47 species of the *Tobamovirus* genus. Potato virus X CP was used as the out-group sequence. CPs are shown relative to the families of the host plants. CPs marked with orange, light blue, and dark green are encoded by the viruses that primarily infect host plants of Solanaceae, Brassicaceae, and Cucurbitaceae, respectively. CPs marked with red and dark blue are encoded by the viruses that can also infect plants in additional families besides Solanaceae and Brassicaceae, respectively. The neighbor-joining (NJ) method was adopted using MEGA software (version 10.1.8) with 1,000 bootstrap replicates. The trees are drawn to scale, with branch lengths measured in the number of substitutions per site. Scale bars, 0.20 amino acid substitutions per site. The individual CP sequences are identified by the name of the virus and the corresponding Protein ID.

The apparent correlation between the host range and phylogeny of the tobamoviral MPs that confer on the virus its cell-to-cell movement ability (see **Figure 1**) suggests that CPs that confer the long-distance movement ability on the same viruses may also correlate with the main host plant species of these viruses. Thus, we analyzed the phylogeny of CPs of the representative members of all tobamoviruses using the CP of potato virus X as the out-group sequence. **Figure 2** shows that, indeed, most of the CP amino acid sequences clustered into clades corresponding to their host plant species, i.e., 17 Solanaceae-infecting viruses and three Brassicaceae-infecting viruses, etc. Thus, viral factors responsible for the local and systemic movement of tobamoviruses may have evolved these capabilities based on their host preferences. This notion is supported by the reported differences in interactions of CPs of different tobamovirus pathotypes with the resistance L proteins of different pepper species (Tomita et al., 2011).

## Host determinants of local and systemic movement

### Phytohormones

Viruses depend on the host cellular machinery throughout most of their infection cycle, from replication to intercellular spread to survival in the infected tissues. Viral survival largely depends on the innate immune responses of the plant (Zheng et al., 2024). Generally, plant-microbe interactions can be either compatible or incompatible according to their outcomes. Hypersensitive response (HR) is a host defense response in incompatible interactions, inducing rapid programmed cell death (PCD) at the infection site and resulting in a clear boundary between the necrotic lesion tissue and the healthy tissue (Lam et al., 2001; Soosaar et al., 2005), effectively restricting the viral particles in the necrotic tissues and the immediately surrounding cells outside the necrotic zone (Appiano et al., 1977; Soosaar et al., 2005). After HR, systemic acquired resistance (SAR) is activated, and it can last for several weeks (Ryals et al., 1996; Sticher et al., 1997).

SAR generally relies on salicylic acid (SA), an important and well-characterized plant hormone that induces resistance against different pathogens, including viruses (Vlot et al., 2009; Kumar, 2014). During tobamoviral infection, SA is involved in the negative regulation of both the MP-mediated cell-to-cell movement and the CP-mediated long-distance movement (Murphy and Carr, 2002; Venturuzzi et al., 2021). During bacterial infection, the PD closure regulated by the SA-induced pathway is associated with the expression levels of the PD-located protein 5 (PDLP5) in *Arabidopsis* (Wang et al., 2013). In addition, plant mutants in critical genes associated with the SA biosynthesis/signaling, such as *ENHANCED DISEASE RESISTANCE1* (*EDS1*), *NONEXPRESSOR OF PATHOGENESIS-RELATED GENES1* (*NPR1*), and *ISOCHORISMATE SYNTHASE1 (ICS1)*, or mutants in the *PDLP5* gene fail to induce deposition of callose (Lee et al., 2011; Wang et al., 2013), the classical negative regulator of PD permeability (Radford et al., 1998; Liu et al., 2020). Consistently, exogenous or endogenous SA induces PDLP5 expression, resulting in callose deposition (Wang et al., 2013). However, SA cannot directly regulate the PD SEL during viral infection, and the molecular mechanisms of the SA effects on tobamoviral movement remain elusive (Murphy et al., 2020).

Besides SA, other phytohormones, including jasmonic acid (JA) and abscisic acid (ABA), may affect the spread of tobamoviruses. The methyl ester of JA (methyl jasmonate, MeJA) enhances the local spread of TMV indirectly by inhibiting the *N* gene that confers HR to TMV (Oka et al., 2013). ABA restricts the viral spread by inducing the synthesis and deposition of callose by regulating its biosynthetic pathway (Kumar and Dasgupta, 2024). Other important phytohormones, such as ethylene and auxin, are important for plant-virus interactions (Knoester et al., 1995; Kalapos et al., 2021; Müllender et al., 2021), but their role in regulating the tobamovirus spread is largely unknown.

### Reactive oxygen species

ROS play a crucial role in response to various abiotic and biotic stresses, especially in sensing and defending against pathogen infection (Mittler et al., 2022). ROS mainly accumulates in chloroplasts, peroxisomes, and mitochondria. However, it can also be produced in the plasma membrane, the endoplasmic reticulum, and the apoplast (Alazem and Burch Smith, 2024); furthermore, the types of ROS may vary due to different stresses (Mittler et al., 2022). Infection by tobamoviruses modulates the ROS signaling pathway, affecting the host immune responses and symptom severity. In addition, specifically for tobamoviruses, their CP can induce ROS accumulation in the infected tissues (Allan et al., 2001; Conti et al., 2017).

ROS targets callose deposits, regulating PD permeability and, therefore, interfering with the movement of tobamoviruses from cell to cell (Alazem and Burch Smith, 2024). ROS controls PD permeability through the interplay of two opposing activities, callose deposition and callose degradation. This is achieved by three pathways: (i) ROS acts as a stress signal and activates callose synthases, e.g., CalS/GSLs, or callose degrading enzymes, e.g., β‐1,3‐glucanases (Cui and Lee, 2016; Alazem and Burch Smith, 2024); (ii) ROS synergistically interacts with PD-associated proteins, e.g., PDLP1 and possibly PDLP5, to change the PD permeability (Fichman et al., 2021); and (iii) ROS functions as a secondary messenger, eliciting an influx of calcium ions, thereby altering the size-exclusion limit of the PD channel (Holdaway-Clarke et al., 2000; Evans et al., 2016; Alazem and Burch Smith, 2024). Interestingly, although the interactions between the SA and ROS signaling pathways are complex, employing both synergistic and antagonistic mechanisms, they regulate the PD permeability independently of each other (Cui and Lee, 2016).

### Host cell cytoskeleton

Generally, plant RNA viruses, including tobamoviruses, are thought to hijack the host cytoskeleton for replication and movement (He et al., 2023). The requirement for intact microfilaments varies between different tobamoviral species; for example, the 125-kDa protein of TVCV forms numerous cytoplasmic microfilament-associated inclusions while its 126-kDa homolog encoded by TMV does not when tested using latrunculin B as a microfilament inhibitor, indicating that functional microfilaments are involved in the movement of TMV but may not be required for the movement of TVCV (Harries et al., 2009). Actin filaments act in association with their molecular motors, myosins (Lee and Liu, 2004). Thus, myosins are also involved in the TMV cell-to-cell movement. Specifically, in *Arabidopsis*, three class VIII myosins and two class XI myosins are required for PD localization and intercellular movement of TMV, respectively (Amari et al., 2014).

TMV MP associates with the ER membrane yet does not integrate into it (Peiró et al., 2014). Whereas targeting TMV MP to PD requires the actin/ER network as indicated using latrunculin and cytochalasin as inhibitors of the actin polymerization (Wright et al., 2007), the PD targeting process likely does not involve the ER-to-Golgi transport as it is not susceptible to brefeldin A (Tagami and Watanabe, 2007; Wright et al., 2007). However, the ER-to-Golgi secretory pathway is required for the intracellular trafficking in several viruses of other genera, including melon necrotic spot virus (MNSV) (Genovés et al., 2010), and Chinese wheat mosaic virus (CWMV) (Andika et al., 2013), etc. This requirement may reflect the need for these viral MPs to form ER-derived structures to maintain their movement or PD targeting functions (Andika et al., 2013). The involvement of microtubules in the tobamoviral MP action, however, remains unresolved. Specifically, the interaction of microtubules with MP appears to regulate the cell-to-cell movement of the viral RNA (Boyko et al., 2000a) and also fulfill an alternative role of targeting MP to degradation (Gillespie et al., 2002).

### Host proteins that interact with tobamoviral MPs

During their cell-to-cell travel, the molecules of tobamoviral MP interact with numerous cellular factors, many of which have been identified, although their specific functions in the movement process often remain to be elucidated. Here, we will summarize tobamovirus MP interactors with known effects on cell-to-cell movement.

In *Arabidopsis*, synaptotagmin A (SYTA) interacts with tobamoviral MPs, potentially binding to their PD-localization sequences, suggesting a hypothesis that SYTA positively regulates the TMV MP cell-to-cell movement via the process of endocytosis or the endocytic recycling pathway (Lewis and Lazarowitz, 2010; Uchiyama et al., 2014; Yuan et al., 2018; Jovanović et al., 2023). In addition, SYTA is required for the ER-PM contacts, which are likely remodeled during the SYTA-MP interaction to supply the replication sites for the virus movement (Levy et al., 2015). Ankyrin repeat-containing protein ANK also interacts with TMV MP, and this interaction was suggested to downregulate callose deposits at PD, increasing PD permeability and facilitating movement (Ueki et al., 2010). Another MP-interacting protein is WPRb, a member of the WEAK CHLOROPLAST MOVEMENT UNDER BLUE LIGHT 1 (WEB1) and PLASTID MOVEMENT IMPAIRED 2 (PMI2)-related protein family. WPRb is involved in the viral cell-to-cell movement by regulating the permeability of PD (Kodama et al., 2011; Cai et al., 2023) by an as yet unknown mechanism. Pectin methylesterases, important plant cell wall proteins, interact with MP and are required for both local and systemic transport of tobamoviruses, and transgenic expression of pectin methylesterase inhibitors restricts virus movement in plants (Dorokhov et al., 1999; Chen et al., 2000; Chen and Citovsky, 2003; Lionetti et al., 2014).

Calreticulin, a highly conserved Ca^2+^-binding protein located at PD (Baluska et al., 1999; Chen et al., 2005; Jia et al., 2009), can interact with TMV MP and influence its subcellular localization and intercellular movement. In addition, overexpression of calreticulin may lead to the mislocalization of MP to microtubules and largely restrict the cell-to-cell movement of MP (Chen et al., 2005).

*Capsicum annuum* HEAT SHOCK PROTEIN26.5 (CaHsp26.5) can induce plant resistance against RNA viruses through interaction with a transcription factor NAC DOMAIN-CONTAINING PROTEIN 81 (ATAF2) (Foong and Paek, 2020). Paradoxically, the loss of function of CaHsp26.5, although compromising the defense response, also restricts the replication and spread of some tobamoviruses, such as TMV and pepper mild mottle virus (PMMoV) (Foong and Paek, 2020). Consistent with this effect, CaHsp26.5 can interact with the MPs of these tobamoviruses in plant cells (Foong and Paek, 2020).

In tomato, a coiled-coil-nucleotide binding site-leucine-rich repeat protein (CC-NLR) encoded by the *Tobacco mosaic virus resistance-2^2^ (Tm-2^2^)* gene represents a durable resistance trait that has been employed for tobamovirus control for many decades. This CC-NLR associates with the tobamoviral MPs (Meshi et al., 1989; Hak and Spiegelman, 2021), and mutations of two amino acids, C68F, and E133K, in TMV MP enable the virus to overcome the *Tm-2^2^* resistance (Meshi et al., 1989). Furthermore, ToBRFV can overcome all known resistance genes in tomato plants, including *Tm-2^2^*, thus emerging as a global threat to tomato crops, and this resistance-breaking ability was reported to associate with the ToBRFV MP, specifically with its 216 amino acid-long N-terminal domain (Hak and Spiegelman, 2021; Salem et al., 2023). Interestingly, replacing ToMV MP with ToBRFV MP enabled the resulting chimeric virus to break the *Tm-2^2^* resistance, although the systemic infection of this viral chimera was limited most likely due to the decrease in the cell-to-cell-movement (Hak and Spiegelman, 2021).

Finally, tobamoviral MPs also interact with and are phosphorylated by protein kinases (Citovsky et al., 1993; Matsushita et al., 2000; Karpova et al., 2002; Matsushita et al., 2003; Yoshioka et al., 2004; Lee, 2008). In the case of TMV MP, phosphorylation has been proposed to regulate its function and movement (Kawakami et al., 1999; Waigmann et al., 2000). Furthermore, BAM1, a receptor-like kinase encoded by *Arabidopsis* and *N. benthamiana*, interacts with MP at PD and facilitates its cell-to-cell transport and the virus spread in the host plants (Tran and Citovsky, 2021).

### Innate immunity

Pathogen-Associated Molecular Pattern (PAMP)-triggered immunity (PTI) and effector-triggered immunity (ETI) are the two well-known layers of the innate immune system in plants against various pathogens (Jones and Dangl, 2006; Boller and He, 2009), including tobamoviruses (Niehl et al., 2016; Zheng et al., 2024). The main plant defense against invading tobamoviruses is RNA silencing (Lopez-Gomollon and Baulcombe, 2022; Huang et al., 2023). To combat this line of defense, plant viruses evolved to encode proteins that act as RNA silencing suppressors (VSR). For example, in tomato mosaic virus (ToMV) and oilseed rape mosaic virus (ORMV), the 130-kDa and 125-kDa replicase-associated protein, respectively, small replication subunit acts as a suppressor of the post-transcriptional gene silencing (Kubota et al., 2003; Csorba et al., 2007; Vogler et al., 2008; Kørner et al., 2018). Conversely, in the absence of VSRs, siRNA molecules with antivirus functions would move systemically ahead of the virus, thus restricting the viral infection (Burgyán and Havelda, 2011). Yet, the relationship between VSR and the viral movement is not fully understood; e.g., in citrus leprosis virus C (CiLV-C), three proteins, p29, p15, and p61 are involved in RNA silencing suppression, and only p15 plays a role in the cell-to-cell viral movement (Leastro et al., 2020).

RNA silencing, as well as PTI, are induced by dsRNA, which represents the replication intermediate of many RNA viruses (Niehl et al., 2016; Lopez-Gomollon and Baulcombe, 2022; Zheng et al., 2024). dsRNA has been proposed to act as a PAMP and induce PTI that targets PD and reduces their permeability by inducing callose deposition, which in turn inhibits the virus cell-to-cell movement (Huang et al., 2023; Zheng et al., 2024). Interestingly, this dsRNA-induced PTI response can be suppressed by MP, indicating that MP may facilitate virus movement by suppressing antiviral defenses (Huang et al., 2023).

### Abiotic factors

Environmental abiotic factors, including temperature, water conditions, and heavy metals, may also affect the spread of tobamoviruses (Boyko et al., 2000b; Prasch and Sonnewald, 2013; Rahman et al., 2021). For example, high temperature increases the association of MP with microtubules, disables the plant defense responses to TMV, and facilitates viral spread (Boyko et al., 2000b; Zhu et al., 2010). Also, non-toxic levels of cadmium ions, one of the major environmental heavy metal pollutants, restrict TVCV systemic spread and block viral disease; interestingly, this loss of systemic transmission was not observed in the presence of high cadmium concentrations (Ghoshroy et al., 1998). The effects of low cadmium concentrations on tobamoviral systemic movement may involve a glycine-rich protein, cdiGRP—the expression of which is induced by low concentrations but not by high concentrations of cadmium ions—that enhances the deposition of callose in the plant vasculature, thus blocking the systemic movement of TVCV (Ueki and Citovsky, 2002).

## Concluding remarks and prospects for future research

**Figure 3:**
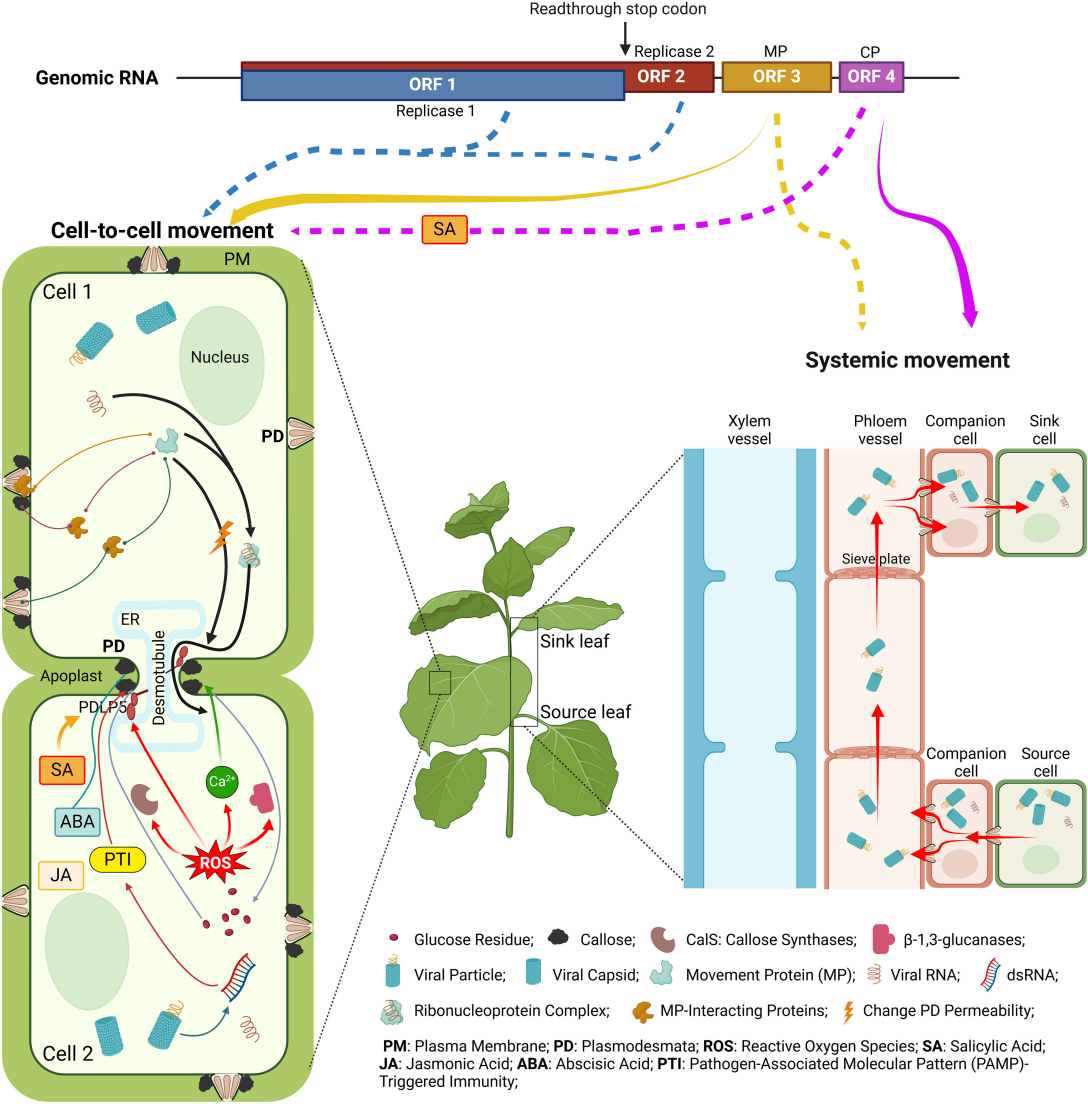
Cell-to-cell and systemic movement of tobamoviruses and the viral and host determinants of these processes. The solid arrows below the genome diagram indicate the classical cell-to-cell and systemic movement pathways facilitated by MP (yellow) and CP (purple), and the dotted arrows point to the involvement of the indicated viral proteins in both types of movement. For descriptions of the specific depicted events, see text. The diagram was constructed from images created using BioRender.com.

Historically, tobamoviruses represent the genus, of which, TMV, was the first virus discovered, laying the foundation for the science of virology. Tobamoviruses, therefore, are one of the most studied groups of plant viruses. Yet our knowledge about one of their critical biological activities, the ability to move between cells and organs of the host plant, remains incomplete. Recent experimentation has provided new insights into these fascinating aspects of the plant-pathogen interaction. Here, we summarized this new information in the context of the fundamental facts that underlie our understanding of the local, cell-to-cell, and systemic, organ-to-organ, movement of tobamoviruses (**Figure 3**). Specifically, we focused on the viral effectors that mediate these transport pathways, MP and CP, respectively. Our phylogenetic analysis of all known amino acid sequences of tobamoviral MPs and CPs revealed that they cluster into distinct clades according to the main host plant family that these viruses naturally infect. Potentially, these phylogenetic differences between MPs and CPs of different tobamoviruses may contribute to their recently recognized broad host range (Zamfir et al., 2023). The cell-to-cell and systemic tobamoviral transport and its MP and CP effectors are described with regard to the MP and CP activities and interactions with different host cell proteins involved in the transport pathway. The roles of the global host cell systems, such as the cytoskeleton, hormonal and ROS responses, and RNA silencing, on the MP- and CP-mediated transport are discussed. Finally, the effects of abiotic factors, such as temperature and heavy metal cadmium, are described, suggesting that anthropogenic climate changes and environmental pollution might alter the ability of tobamoviruses, and most likely other plant viruses, to move within the infected plants.

What are the important challenges in the field of tobamoviral transport for the near future? One such endeavor would be assembling a jigsaw puzzle of numerous MP and CP interactors identified to date into a meaningful and comprehensive model of the transport pathway(s) with a clear function for each interactor in the positive and negative control of the movement process. A more long-term challenge could lie in uncovering potential mechanistic connections between the MP and CP sequences and the host plant preference of the virus. Without a doubt, the research toward these goals will be substantially facilitated by emerging technologies, such as AI-based protein modeling trained on large datasets of sequences already available for plants to unravel protein interaction models, cryo-electron tomography to enhance 3D imaging of PD structure, 3D images of frozen biological samples, using the proximity-based bioluminescence techniques to detect interactions between viral and host proteins interactions in real-time, etc. We believe that data from these lines of research will provide novel and exciting insights into plant virology, continuing the reputation of tobamoviruses, specifically TMV, as viruses of “many firsts” (Creager et al., 1999; Spiegelman and Dinesh-Kumar, 2023).
